# *Leishmania infantum*, *Dirofilaria* spp. and other endoparasite infections in kennel dogs in central Italy

**DOI:** 10.1051/parasite/2018001

**Published:** 2018-02-01

**Authors:** Federica Sauda, Livia Malandrucco, Gladia Macrì, Manuela Scarpulla, Claudio De Liberato, Giuliana Terracciano, Gianluca Fichi, Federica Berrilli, Stefania Perrucci

**Affiliations:** a Dipartimento di Scienze Veterinarie-Università di Pisa, Pisa Italy; b Ospedale Veterinario ASL Roma D, Via della Magliana 856, Rome Italy; c Istituto Zooprofilattico Sperimentale del Lazio e della Toscana M. Aleandri (IZSLT), Rome and Pisa Italy; d Dipartimento di Medicina Sperimentale e Chirurgia, Università degli Studi di Roma Tor Vergata, Rome Italy

**Keywords:** Kennel dogs, Endoparasite infections, Prevalence, Risk factors, Zoonosis

## Abstract

Prevalence and risk factors of *Leishmania infantum*, *Dirofilaria* spp. and other potentially zoonotic or canine-specific endoparasite infections were assessed in 639 kennel dogs from central Italy. To this end, individual blood and fecal samples were examined using parasitological, immunological and molecular techniques. The presence of compatible clinical pictures, as well as age and gender were considered as putative risks factors. To evaluate risk factors, multivariable analysis with logistic regression and univariable analysis with a Chi square test and a Fischer’s exact test were performed. Overall, 52.6% of dogs (95% CI 48.6-56.5) were found positive, while 39.6% of dogs (95% CI 35.8-43.5) were infected by potentially zoonotic species. *Leishmania infantum and* *Dirofilaria repens* showed prevalences of 2.5% (95% CI 1.5-4.1) and 2.8% (95% CI 1.7-4.5), respectively. The prevalence of cardiorespiratory parasites was 7.8% (95% CI 5.9-10.3) and included the species *Angiostrongylus vasorum*, *Eucoleus aerophilus*, *Eucoleus boehmi* and *D. immitis*; the latter showed a prevalence of 0.2% (95% CI 0.001-1). Intestinal parasites were significantly prevalent (38.8%, 95% CI 35-42.7) and they consisted mainly of species of major zoonotic concern, including ancylostomatids, *Toxocara canis*, *Giardia duodenalis*, *Dipylidium caninum*, Taeniidae, *Strongyloides stercoralis* and *Cryptosporidium parvum*. Endoparasites were significantly prevalent in clinically suspected dogs. *Leishmania infantum* and cardiorespiratory nematodes were prevalent in older dogs, while intestinal parasites were prevalent in younger dogs. Results show high dog and public health risks in kennels in central Italy, and suggest the need for more effective control measures.

## Abstract (fr)

La prévalence et les facteurs de risque de *Leishmania infantum*, *Dirofilaria* spp. et d’autres infections à endoparasites potentiellement zoonotiques ou spécifiques aux chiens ont été évalués chez 639 chiens de chenils du centre de l’Italie. À ces fins, des échantillons individuels de sang et de selles ont été examinés par des techniques parasitologiques, immunologiques et moléculaires. La présence d’images cliniques compatibles, l’âge et le sexe ont été considérés comme des facteurs de risque putatifs. Pour l’évaluation des facteurs de risque, une analyse multivariée avec une régression logistique et une analyse univariée avec un test de Chi carré et un test exact de Fischer ont été réalisées. Dans l’ensemble, 52,6% (IC à 95%: 48,6-56,5) des chiens ont été trouvés positifs, tandis que 39,6% des chiens (IC à 95%: 35,8-43,5) étaient infectés par des espèces potentiellement zoonotiques. *Leishmania infantum* et *Dirofilaria repens* ont montré respectivement des prévalences de 2,5% (IC 95% 1,5-4,1) et 2,8% (IC 95% 1,7-4,5). La prévalence de parasites cardiorespiratoires était de 7,8% (IC 95% 5,9-10,3) et comprenait les espèces *Angiostrongylus vasorum*, *Eucoleus aerophilus*, *Eucoleus boehmi* et *Dirofilaria immitis* ; cette dernière avait une prévalence de 0,2% (IC 95% 0,001-1). Les parasites intestinaux étaient significativement prévalents (38,8%, IC 95% 35-42,7) et consistaient principalement en espèces zoonotiques majeures, dont les Ancylostomatidae, *Toxocara canis*, *Giardia duodenalis*, *Dipylidium caninum*, les Taeniidae, *Strongyloides stercoralis* et *Cryptosporidium parvum*. Les endoparasites étaient significativement prévalents chez les chiens cliniquement suspectés. *Leishmania infantum* et les nématodes cardiorespiratoires étaient répandus chez les chiens plus âgés, alors que les parasites intestinaux étaient répandus chez les chiens plus jeunes. Les résultats montrent des risques élevés pour les chiens et la santé publique dans les chenils du centre de l’Italie et suggèrent la nécessité de mesures de contrôle plus efficaces.

## Introduction

In kennel dogs, the prevalence of endoparasite infections is frequently high and may include potential zoonotic and significantly virulent species, often resulting in high dog and public health risks [14,26,28,44]. According to current Italian legislation, stray dogs can be euthanized only when found to be affected by severe diseases or proven to be dangerous for human safety. In all other cases, Italian legislation requires housing of caught stray dogs in public and private kennels where they remain throughout their lives, unless private owners adopt them [18,45]. Therefore, in Italian dog kennels animal density is often very high and may pose serious risks for the spread of canine-specific and potential zoonotic parasites [29,53].

Among dog parasites of major zoonotic concern, *Leishmania infantum* is an important vector-borne parasite of the dog, which is considered the main domestic reservoir host for human infections [34]. Canine leishmaniosis is endemic in Italy and in other countries of the Mediterranean basin, with a widely variable prevalence [15,40].

*Dirofilaria immitis* and *Dirofilaria repens* are two vector-borne dog filariid nematodes that have been recognized as emerging zoonotic agents, currently spreading throughout Europe [11,14,24,30,43]. In dogs, *D. repens* is the etiologic agent of subcutaneous dirofilariosis, while *D. immitis* is the cause of cardiopulmonary dirofilariosis [14].

Infections by respiratory parasites are common in kennel dogs and include the species *Eucoleus aerophilus*, a potentially zoonotic nematode [9,51].

Concerning intestinal parasites, they may reach a prevalence of up to about 70% in kennel dogs [4,28,44]. Potential zoonotic intestinal parasites of dogs include *Toxocara canis*, the agent of ocular and visceral *larva migrans* in humans and of lung and intestinal diseases in dogs [20]. Dog hookworms (mainly *Ancylostoma* spp.) may be responsible for severe intestinal disease in dogs and cutaneous *larva migrans* or creeping eruption and eosinophilic enteritis in humans [5,10,31]. Although *Strongyloides stercoralis* is considered a potential zoonotic nematode parasite of dogs, and human infections have been related to infected dog exposure, recent molecular studies have shown that there are clear differences between human and dog strains and the existence of *S. stercoralis* subspecies has been proposed. Therefore, the zoonotic potential of dog *S. stercoralis* is now being challenged and further studies are currently considered of high importance to clarify this serious issue [50]. The tapeworms *Echinococcus* spp. and *Dipylidium caninum* are further dog intestinal helminths of zoonotic concern [44]. Zoonotic genotypes of the protozoa *Giardia duodenalis* and *Cryptosporidium* spp. may be shared by dogs and humans, in both these species they may be responsible for intestinal signs of variable severity [38,47].

With the aim of verifying the effectiveness of preventive and control measures taken in Italian kennels for dog endoparasites, with particular reference to potentially zoonotic species, this study evaluated the occurrence, prevalence and potential risk factors of *L. infantum*, *D. repens* and of potentially zoonotic or host-specific cardiorespiratory and intestinal parasite infections in kennel dogs in central Italy.

## Materials and methods

### Ethics statement

Collection of samples and manipulation of animals were authorized by the kennels and the Italian Ministry of Health in the framework of the Italian surveillance programs of potential zoonotic diseases of stray animals (Italian law No. 281-1991). Moreover, veterinarians who performed the sampling and who handled animals examined in this study were authorized by the Italian Ministry of Health.

### Animals

In the period November 2011 − November 2014, a total of 639 randomly selected dogs of different breeds and living in public and private kennels of Latium and Tuscany (central Italy), were examined in order to assess the prevalence of dog endoparasite infections caused by *L. infantum, D. repens*, intestinal and cardiorespiratory parasites. All facilities are equipped with green or gravel common areas where dogs can move freely, while dog density was about 500 animals per kennel as a mean (range 100-800 dogs). In all these kennels, animals are treated with anthelmintic drugs at the time of their entry and then about once or, more rarely, twice a year. Ectoparasiticide treatments are performed only if dogs are found infected by ectoparasites at their entry.

No dogs included in the study were vaccinated against *L. infantum* or had received chemoprophylaxis for cardiopulmonary dirofilariosis.

All animals were clinical examined in order to evaluate possible presence of compatible clinical pictures.

Among the examined dogs, 538/639 were males and 101/639 were females, while regarding the age 395/639 dogs were ≤ 24 months and 244/639 dogs were older than 24 months.

### Sampling

Individual blood and fecal samples were collected from all examined dogs. From each dog, at least 3 mL of blood were collected, *i.e.* 1 mL in tubes with EDTA and 2 mL in tubes without anticoagulant. All blood samples were stored at 4 °C and analyzed within 24 hours. Blood samples without anticoagulant were centrifuged at 3000 rpm for 10 min to obtain sera that were used for the immunological analysis of *D. immitis* and *L. infantum*, while blood samples diluted in EDTA were analyzed to detect blood-circulating microfilariae.

An aliquot of each fecal sample was frozen at −20 °C, pending further molecular analysis. Fecal samples (about 5 g) were stored at 4 °C and analyzed within 24 hours.

### Microscopic examination of blood samples for detection of circulating microfilariae

Blood samples in EDTA were tested using the modified Knott technique for the detection of blood-circulating microfilariae that were identified at the species level based on the morphology of the head and tail, length and width [46,51].

### Detection of *D. immitis* circulating antigens

For the detection of *D. immitis* circulating antigens, serum samples were tested using a canine antigen test kit (PetChek HTWM PF, IDEXX, Westbrook, Maine, USA) according to the manufacturer’s instructions .

### Serology for *Leishmania infantum*

Serum samples were analyzed by an indirect fluorescent antibody test (IFAT) to investigate the presence of specific antibodies against *L. infantum*.

The in-house IFAT was performed as described in the Manual of the World Organization for Animal Health [27]. The antigen was prepared with promastigotes of *L. infantum* MHOM/TN/80/IPT1, WHO reference strain of *L. infantum*, provided by the Istituto Superiore di Sanità (Rome, Italy) and cultured in the IZSLT laboratory (Rome, Italy). Anti-*Leishmania* antibodies were detected using anti-dog IgG conjugated to fluorescein isothiocyanate (Sigma-Aldrich, St. Louis, Missouri, USA). Positive dog serum samples were used as positive controls, while negative dog serum samples and wells containing the antigen and PBS instead of dog serum were used as negative controls. Samples were classified as positive if promastigote cytoplasmic or membrane fluorescence was observed at a serum dilution ≥ 1:160. Reading was performed with an immunofluorescence microscope independently by two observers.

### Fecal analysis

Samples were examined for parasites by macroscopic examination to detect the presence of proglottids, nematodes and/or fragments of parasites and then microscopically by flotation test with a low density solution (saturated NaCl solution, s.g. 1.2), to evaluate the presence of worm eggs and/or protozoal (oo)cysts [36]. Fresh and Lugol-stained fecal smears were also prepared to detect *G. duodenalis*. Moreover, a commercial rapid immunoassay (RIDA QUICK *Cryptosporidium/Giardia* Combi, R-Biopharm^®^, Darmstadt, Germany) was used to detect *G. duodenalis* and *Cryptosporidium* fecal antigens.

Selected *Giardia*-positive samples were processed by a commercial kit (QIAamp DNA Stool Mini Kit, QIAGEN, Valencia, California, USA) for DNA extraction. A nested PCR protocol was applied to amplify a fragment of the small subunit ribosomal RNA (*SSUrDNA*) gene. For external PCR, the forward primer RH11 (5′-CATCCGGTCGATCCTGCC-3′) and the reverse primer RH4 (5′-AGTCGAACCCTGATTCTCCGCCCAGG-3′) were used; the internal primers were GIAR-F forward (3′-GACGCTCTCCCCAAGGAC-5′) and GIAR-R reverse (5′-CTGCGTCACGCTCG-3′) [35]. Amplification products were run on 2% ethidium bromide agarose gels and visualized under ultraviolet light. Positive amplicons were purified using a QIAquick Gel Extraction Kit (QIAGEN, Valencia, California, USA). Amplification products were sent to an external laboratory for sequencing (Bio-Fab Research, Rome, Italy); sequence multiple alignment was carried out by ClustalW to identify *G. duodenalis* assemblages. To identify *G. duodenalis* assemblages, sequence multiple alignment was carried out by ClustalW against reference sequences available in GenBank representing *Giardia* assemblages A-G [Accession Numbers: AF199446 (assemblage A), AF199447 (assemblage B), AY775200 (assemblage C), AY775199 (assemblage D), AY297957 (assemblage E), AF199444 (assemblage F) and AF199450 (assemblage G)].

DNA of *Cryptosporidium* spp. was extracted from stool samples found positive on rapid immunoassay by using a QIAamp DNA stool mini kit (QIAGEN, Valencia, California, USA), following the manufacturer’s instructions modified by the European Union Reference Laboratory for Parasites, Istituto Superiore di Sanità (Rome, Italy).

An RFLP-PCR protocol was applied to amplify fragments of the COWP *Cryptosporidium* spp. gene and to identify *Cryptosporidium* at the species level [33]. For the nested-COWP procedure (N-COWP), an external 769-bp fragment of the COWP gene was amplified with forward primer BCOWPF (5’-ACCGCTTCTCAACAACCATCTTGTCCTC-3’) and reverse primer BCOWPR (5’-CGCACCTGTTCCCACTCAATGTAAACCC-3’). For the internal 553-bp COWP gene fragment, the primers Cry9 (5’-GGA CTG AAA TAC AGG CAT TAT CTT G-3’) and Cry15 (5’-GTA GAT AAT GGA AGA GAT TGT G-3’) were used [30,44]. Amplification products were run on 2% agarose gels containing Gel Red 10,000X (Biotium; Hayward, California, USA) and visualized under ultraviolet light. *Rsa*I digestion of N-COWP fragments was resolved by electrophoresis in 3% agarose gels containing Gel Red 10,000X (Biotium; Hayward, California, USA) [7]. The number and size of restriction fragments were used to identify the species of *Cryptosporidium*, as previously described [30,47,48]. Positive and negative controls were included in each *Giardia/Cryptosporidium* PCR and nested PCR run.

For the isolation of nematode larvae from fecal samples, such as *A. vasorum* and *S. stercoralis* larvae, the Baermann technique was used, and isolated larvae were identified according to their morphological and metric features [51].

### Statistical methods

A database was developed *ad hoc* using Excel 5.0. Data analysis was performed using the statistical software Epi Info Version 3.5.3.

Gender, age and presence of compatible clinical pictures were the putative risk factors considered in the study. To evaluate risk factors, multivariable analysis with logistic regression and a Chi square test for univariable analysis were performed. The Fischer’s exact test was used when the percentages were small. The significance level was set at *p* < 0.05.

## Results

An overall prevalence of 52.6% (336/639) (95% CI 48.6-56.5) was observed in examined animals for investigated endoparasite infections. Moreover, 253/639 dogs (39.6%) (95% CI 35.8-43.5) were found infected by potentially zoonotic species and 249/639 dogs (39%) (95% CI 35.2-42.9) showed compatible clinical pictures (Table 1).

**Table 1:** Prevalence and 95% confidence interval (CI) of intestinal, cardiorespiratory and vector-borne parasites identified in the total number of examined dogs (639) and in the same dogs separated into apparently healthy or clinically suspect dogs on the basis of clinical examination.

	Total dogs	Apparently healthy dogs	Clinically suspected dogs
Intestinal helminths	27.5% (176/639) (95% CI 24.1-31.2)	23.9% (42/176) (95% CI 17.8-30.9)	76.1% (134/176) (95% CI 69.1-82.2)
Intestinal protozoa	11.3% (72/639) (95% CI 9-14)	2.8% (2/72) (95% CI 0.3-9.7)	97.2% (70/72) (95% CI 90.3-99.7)
Cardiorespiratory nematodes	7.8% (50/639) (95% CI 5.9-10.3)	32% (16/50) (95% CI 19.5-46.7)	68% (34/50) (95% CI 53.3-80.5)
*Dirofilaria repens*	2.8% (18/639) (95% CI 1.7-4.5)	94.5% (17/18) (95% CI 72.7-99.9)	5.5% (1/18) (95% CI 0.1-27.3)
*Leishmania infantum*	2.5% (16/639) (95% CI 1.5-4.1)	37.5% (6/16) (95% CI 15.2-64.6)	62.5% (10/16) (95% CI 35.4-84.8)

As shown in Table 1, *L. infantum* and *D. repens* had a prevalence of 2.5% (16/639, 95% CI 1.5-4.1) and 2.8% (18/639, 95% CI 1.7-4.5), respectively. In addition, *L. infantum* was found significantly prevalent in dogs older than 2 years in age (*p* < 0.05).

The overall prevalence of cardiorespiratory nematodes was 7.8% (50/639, 95% CI 5.9-10.3), but these infections were found significantly prevalent (*p* < 0.05) in older dogs (age ≥ 2 years). Identified species included *Angiostrongylus vasorum* (5.2%, 95% CI 3.6-7.3), *Eucoleus aerophilus* (1.7%, 95% CI 0.9-3.2), *Eucoleus boehmi* (0.8%, 95% CI 0.3-1.9) and *D. immitis* (0.2%, 95% CI 0.001-1).

Intestinal parasites were prevalent (38.8%, 248/639, *p* < 0.05) in examined dogs and the age ≤ 2 years was found to be a risk factor for intestinal parasite infections (*p* < 0.05). Identified intestinal parasites included ancylostomatids (*A. caninum* and *Uncinaria stenocephala*) (11.3%, 95% CI 9-14), *T. canis* (7.8%, 95% CI 5.9-10.3), *Trichuris vulpis* (7.5%, 95% CI 5.6-9.9), *Cystoisospora canis* (5.9%, 95% CI 4.3-8.10), *Giardia duodenalis* (4.8%, 95% CI 3.4-6.9), *D. caninum* (0.5%, 95% CI 0.1-1.5), Taeniidae eggs (0.3%, 95% CI 0.1-1.3), *Entamoeba* sp. (0.3%, 95% CI 0.1-1.3), *S. stercoralis* (0.2%, 95% CI 0.0.001-1) and *Cryptosporidium* spp. (0.2%, 95% CI 0.001-1).

In molecular studies, the assemblages A (33%, 95% CI 16.7-51.4) and C (67%, 95% CI 48.6-83.3) of *G. duodenalis* and the species *Cryptosporidium* *parvum* were identified.

In the statistical analysis, investigated endoparasite infections were overall found significantly associated with the presence of compatible clinical pictures (*p* < 0.05). The main clinical signs found in animals positive for intestinal helminth infections were diarrhea (41.4%), polyphagia (19.3%) and weight loss (15.3%), while diarrhea was the prevalent clinical sign (97.2%) in dogs affected by intestinal protozoa. Symptomatic dogs affected by cardiorespiratory parasite infections showed cough (23.5%), nasal discharge (17.6%), reverse sneezing (8.8%), heart failure (8.8%), and dyspnea (5.8%). A dog infected by *D. repens* showed skin nodules (5.5%). Finally, symptomatic *L. infantum* infected dogs showed furfuraceous dermatitis with alopecic areas on the face (50%), onychogryphosis (20%), uveitis (10%), lameness (10%), and weight loss (10%).

Coinfections were found in 3.9% (25/639, 95 % CI 2.6-5.8) of examined dogs, and males (84%, 21/25; 95% CI 63.9-95.5) were found more frequently infected by different parasite species than females (16%, 4/25; 95% CI 4.5-36.1) at statistical analysis (*p* < 0.05).

## Discussion

Results from this study add new and significant data on the prevalence of *L. infantum, Dirofilaria* spp. and other endoparasite infections in kennel dogs in Italy. First, the results obtained show a significant prevalence of these parasites in this dog population as more than half of the examined animals were positive for at least one parasite species. Second, about 4/10 dogs were found to be infected by potentially zoonotic species. These findings confirm the important role played by kennel and shelter dogs in Italy in the spread of potential zoonotic parasites [26,28,31,44,53]. As already reported [44], results from the present study are also indicative of high health risks for dogs housed in these facilities, especially considering that among positive dogs, examined parasites were significantly prevalent in clinically suspect animals.

In this study, the seroprevalence of *L. infantum* (2.5%) was found to be higher in older animals and overall similar to that recently observed in kennels from other areas of central and northern Italy, showing low/medium infection levels, from 2.8% to 17.9% [3,39,40]. *L. infantum* is a protozoan species responsible for a very important zoonotic disease representing a serious veterinary and public health problem. Transmission of *L. infantum* occurs through phlebotomine sand fly vectors, and dogs are the main domestic reservoirs of this protozoan parasite [3,6,40]. In endemic areas, the identification of infected dogs and the use of effective control measures are considered extremely important for limiting the spread of the disease and interrupting the transmission of *L. infantum* to the vector [6]. This is the main reason why in these areas, leishmaniosis surveillance programs are carried out in dog kennels, with monitoring activities aimed at identifying infected animals [3,39,40]. The parasitological and clinical positivity found in dogs examined in this study underline the need to improve interventions on leishmaniosis surveillance and control in public and private dog kennels in Italy.

Prevalence observed for *D. repens* (2.8%) is in line with data previously reported in kennel dogs from central Italy, ranging from 1.7% to about 12% [21,41]. *D. immitis* is a further filariid species considered the most important cardiopulmonary nematode in dogs [23]. In the present study, *D. immitis* showed a low prevalence (0.2%) since a single dog was found positive at serological and microscopic analysis. This result differs greatly from previous data reported in kennel dogs in Italy where higher prevalence rates (2.8-12.5%) were observed [8,21,47]. However, this result confirms the findings of a previous study in dogs from central Italy [41]. Importantly, both methods used in this study to evaluate the prevalence of *D. immitis* may fail in the identification of some infected dogs, especially those with low *D. immitis* burdens and it is possible that the prevalence found in this study is underestimated. Indeed, dogs with low *D. immitis* numbers may have single sex infections resulting in absence of patency. In areas of high heartworm prevalence, some dogs may also possess anti-microfilaria antibodies through repeated infections that promote clearance of microfilariae from the circulation [19]. Furthermore, antigen detection tests can give false-negative results due to low worm counts, infections with immature worms and all-male infections, since they target the antigens released from the reproductive tract of adult *D. immitis* females [42]. In Europe, *D. repens* is the main agent of human dirofilariosis [17,23], while *D. immitis* human infections have recently been reported in Italy [2]. Considering the zoonotic potential of both these parasite species and the severity of the disease that *D. immitis* may cause in infected dogs, the results obtained here suggest the need to perform effective control measures against these nematodes in kennel dogs, including chemoprophylaxis for cardiopulmonary dirofilariosis.

Besides *D. immitis*, in examined dogs the (cardio)-respiratory nematode species *E. boehmi, E. aerophilus* and *A. vasorum* were also identified. Overall, these parasites were found to be prevalent in older animals. *E. aerophilus* and *E. boehmi* are two closely related capillariid nematodes that live embedded underneath the epithelium of the upper respiratory tract [9,54]. *E. aerophilus* infects the trachea and bronchi and is a potentially zoonotic species [51]. The prevalence of *E. aerophilus* found in the present study (1.7%) is consistent with previous data reported in dogs from Italy, ranging from about 0.3% to 17% [9,31,50,53]. Concerning *E. boehmi*, a canine nasal nematode, the prevalence observed in this study was lower than that (2.2%) previously reported in dogs [22]. *A. vasorum* is a cardiopulmonary nematode whose adults localize in the right heart and the pulmonary artery of the dog and may be responsible for cardiorespiratory and neurologic signs and coagulopathies, with possible fatal outcomes [23,52]. The prevalence of *A. vasorum* found in this study (5%) is higher than that observed (about 2%) in dogs from other areas of Italy [8,9,16]. This finding confirms that regions in central Italy may offer ideal environmental and epidemiological conditions for the spread of this parasite [9,16]. Regarding intestinal species, data from this study show that young age is an important factor associated with a higher prevalence of these parasites in dogs and confirm that these infections, mainly ascarid and ancylostomatid species potentially pathogenic for humans, are prevalent in kennel dogs in Italy [53]. As previously observed [28,31,37,44], data from this study also show that in kennel dogs, intestinal helminths are more prevalent than intestinal protozoa. Nevertheless, *D. caninum, S. stercoralis* and taeniids showed a low prevalence, although these results may be underestimated for the low sensitivity of macroscopic examination and the flotation test to diagnose cestodes [44] and of the Baermann technique to diagnose *S. stercoralis* [32,50]. Concerning intestinal protozoa, in examined dogs *G. duodenalis* was identified with a prevalence (5%) lower than that recently observed in other shelters and kennels in Europe (about 7-16%), including Italy [25,28,31,44,49,53]. With regard to *Cryptosporidium*, results obtained in this study agree with previous data from Italy, showing a prevalence of dog cryptosporidiosis ranging from 0.2% to 3.3% [12,13,31,44,50,55]. However, the identification among positive dogs of *Giardia* (assemblage A) and *Cryptosporidium* (*C. parvum*) genotypes of zoonotic concern, may indicate potential infection risks for human operators working in these kennels and also for other humans if the (oo)cysts of these protozoan parasites can contaminate the environment outside infected rescues. The prevalence of coccidian (*C. canis*) infections found in this study is in line with previous data from owned and kenneled dog populations in Italy, reporting a prevalence of about 6-10% [36,44,50]. Few dogs (0.3%) were found positive for *Entamoeba* spp., a genus that includes a group of intestinal protozoan species of humans and other mammals all over the world [1].

In conclusion, data from this study show high prevalence rates of *L. infantum, D. repens*, and intestinal and cardiorespiratory parasites in kennel dogs of Italy. Prevalence of identified parasites varied mainly according to the age of dogs examined here. However, intestinal and potentially zoonotic species were prevalent. In a context of improving the health and welfare of housed dogs and reducing the risk of disease transmission to humans in kennels in Italy, these results highlight the need to perform more frequent and effective parasitological surveillance, preventive interventions and treatments of infected animals. Effective environmental management procedures and hygiene measures should also be implemented.

## Funding

This research did not receive any specific grant from funding agencies in the public, commercial, or not-for-profit sectors.

## Conflict of interest

The authors declare that they have no conflicts of interest in relation to this article.
